# Supplementary material for: Systematic characterization of gene function in the photosynthetic alga Chlamydomonas reinhardtii
Source: Nat Genet. 2022 May 5;54(5):705–14. doi: 10.1038/s41588-022-01052-9 (PMC9110296; doi:10.1038/s41588-022-01052-9)
Supplement: Supplementary file 1 — List of supplementary materials [file 41588_2022_1052_MOESM1_ESM.pdf]

nature portfolio

https://doi.org/10.1038/s41588-022-01052-9

Supplementary information

Systematic characterization of gene function in the photosynthetic alga *Chlamydomonas reinhardtii*

In the format provided by the authors and unedited

LIST OF SUPPLEMENTARY MATERIALS

Supplementary Table 1 | Source material used for screens

Supplementary Table 2 | List of treatments and screens

Supplementary Table 3 | LATCA screen and dose titration validation

Supplementary Table 4 | Mutant phenotypes across all screens (https://doi.org/10.6086/D1Q96Z)

Supplementary Table 5 | FDRs for GO term enrichment

Supplementary Table 6 | FDRs for all genes in all screens

Supplementary Table 7 | High-confidence gene-phenotype relationships

Supplementary Table 8 | Annotation of 50 gene-phenotype relationships

Supplementary Table 9 | Suggested new gene names

Supplementary Table 10 | Phenotypic and transcriptomic correlations of genes with high-confidence phenotypes

Supplementary Table 11 | Cluster annotations with yeast, mouse, and Arabidopsis orthologs

Supplementary Table 12 | Mutant barcode read counts (https://doi.org/10.6086/D1Q96Z)

Supplementary Table 13 | List of samples that were averaged

Supplementary Table 14 | Chlamydomonas and Arabidopsis strains used in this study

Supplementary Table 15 | Primers used in this study

Supplementary Data 1 | LATCA compound structures

Supplementary Data 2 | Java TreeView files of FDR less than 0.3 gene clusters
