# Supplementary material for: Systematic characterization of gene function in the photosynthetic alga Chlamydomonas reinhardtii
Source: Nat Genet. 2022 May 5;54(5):705–14. doi: 10.1038/s41588-022-01052-9 (PMC9110296; doi:10.1038/s41588-022-01052-9)
Supplement: Supplementary file 2 — Reporting Summary [file 41588_2022_1052_MOESM2_ESM.pdf]

nature research

Corresponding author(s): Robert Jinkerson, Martin Jonikas, Jose Dinneny

Last updated by author(s): 12/13/2021

Reporting Summary

Nature Research wishes to improve the reproducibility of the work that we publish. This form provides structure for consistency and transparency in reporting. For further information on Nature Research policies, see our Editorial Policies and the Editorial Policy Checklist.

Statistics

For all statistical analyses, confirm that the following items are present in the figure legend, table legend, main text, or Methods section.

n/a	Confirmed	
☐	☒	The exact sample size (n) for each experimental group/condition, given as a discrete number and unit of measurement
☐	☒	A statement on whether measurements were taken from distinct samples or whether the same sample was measured repeatedly
☐	☒	The statistical test(s) used AND whether they are one- or two-sided<br>*Only common tests should be described solely by name; describe more complex techniques in the Methods section.*
☒	☐	A description of all covariates tested
☐	☒	A description of any assumptions or corrections, such as tests of normality and adjustment for multiple comparisons
☐	☒	A full description of the statistical parameters including central tendency (e.g. means) or other basic estimates (e.g. regression coefficient) AND variation (e.g. standard deviation) or associated estimates of uncertainty (e.g. confidence intervals)
☐	☒	For null hypothesis testing, the test statistic (e.g. F, t, r) with confidence intervals, effect sizes, degrees of freedom and P value noted<br>*Give P values as exact values whenever suitable.*
☒	☐	For Bayesian analysis, information on the choice of priors and Markov chain Monte Carlo settings
☐	☒	For hierarchical and complex designs, identification of the appropriate level for tests and full reporting of outcomes
☐	☒	Estimates of effect sizes (e.g. Cohen's d, Pearson's r), indicating how they were calculated

Our web collection on statistics for biologists contains articles on many of the points above.

Software and code

Policy information about availability of computer code

Data collection	No software was used for data collection.
Data analysis	Analysis was conducted with Python (2.7.11), SciPy (0.17.0), Pandas (0.18.1), Matplotlib (1.5.1), and Seaborn (0.7.1). Details can be found in the methods. Custom code used for data analysis has been deposited in Zenodo (https://doi.org/10.5281/zenodo.6340170).

For manuscripts utilizing custom algorithms or software that are central to the research but not yet described in published literature, software must be made available to editors and reviewers. We strongly encourage code deposition in a community repository (e.g. GitHub). See the Nature Research guidelines for submitting code & software for further information.

Data

Policy information about availability of data

All manuscripts must include a data availability statement. This statement should provide the following information, where applicable:

- Accession codes, unique identifiers, or web links for publicly available datasets
- A list of figures that have associated raw data
- A description of any restrictions on data availability

All data, including mutant barcode read count data (Supplementary Table 12) and mutant phenotypes across all screens (Supplementary Table 4), can be found at https://doi.org/10.6086/D1Q96Z in the Dryad Digital Repository.

Field-specific reporting

Please select the one below that is the best fit for your research. If you are not sure, read the appropriate sections before making your selection.

☒ Life sciences ☐ Behavioural & social sciences ☐ Ecological, evolutionary & environmental sciences

For a reference copy of the document with all sections, see nature.com/documents/nr-reporting-summary-flat.pdf

Life sciences study design

All studies must disclose on these points even when the disclosure is negative.

Sample size	We empirically determined the sample sizes based on published research.
Data exclusions	Data from screens where the conditions were extremely harsh, such as camptothecin, that resulted in population bottlenecks where the majority of mutants/barcodes are lost were excluded from analysis.
Replication	Replicates of many screens were performed. Most mutants have similar phenotypes in screen replicates (Fig. 2C).
Randomization	Randomization was not applicable for this study.
Blinding	Blinding and randomization were not used for this study.

Reporting for specific materials, systems and methods

We require information from authors about some types of materials, experimental systems and methods used in many studies. Here, indicate whether each material, system or method listed is relevant to your study. If you are not sure if a list item applies to your research, read the appropriate section before selecting a response.

Materials & experimental systems

n/a	Involved in the study	
☐	☒	Antibodies
☐	☒	Eukaryotic cell lines
☒	☐	Palaeontology and archaeology
☐	☒	Animals and other organisms
☒	☐	Human research participants
☒	☐	Clinical data
☒	☐	Dual use research of concern

Methods

n/a	Involved in the study	
☒	☐	ChIP-seq
☒	☐	Flow cytometry
☒	☐	MRI-based neuroimaging

Antibodies

Antibodies used	anti-actin antibody (clone C4, EMD Millipore, MAB1501); HRP-conjugated anti-mouse IgG (ICN Pharmaceuticals; 55564); anti-rabbit IgG (Southern Biotech; 4050-05); rabbit anti-NAP1 antibody (generous gift from Ritsu Kamiya and Takako Kato-Minoura PMID:12796293). The following dilutions were used: Anti-actin, 1:500; Anti-mouse HRP, 1:5000; Anti-NAP1, 1:1000; Anti-rabbit HRP, 1:20,000.
Validation	We observed decreasing abundance of IDA5 upon addition of LatB, conversely we observed increased abundance of NAP1 consistent with these antibodies recognizing their intended targets.

Eukaryotic cell lines

Policy information about cell lines

Cell line source(s)	CC-4533 CW15 MT- [JONIKAS CMJ030] www.chlamycollection.org
Authentication	N/A
Mycoplasma contamination	N/A
Commonly misidentified lines (See ICLAC register)	N/A

April 2020

Animals and other organisms

Policy information about studies involving animals; ARRIVE guidelines recommended for reporting animal research

Laboratory animals	Predators of algae were purchased from Carolina Biologic and cared for based on their recommendations.
Wild animals	N/A
Field-collected samples	N/A
Ethics oversight	No institutional ethical approval was required because the animals were invertebrates (Daphnia, rotifers, and water bears)

Note that full information on the approval of the study protocol must also be provided in the manuscript.
