# Supplementary figures and images for: Systematic characterization of gene function in the photosynthetic alga Chlamydomonas reinhardtii (part 2 of 2)
Source: Nat Genet. 2022 May 5;54(5):705–14. doi: 10.1038/s41588-022-01052-9 (PMC9110296; doi:10.1038/s41588-022-01052-9)

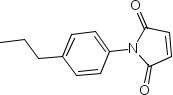

Supplement: Supplementary file 5 — Zip file that contains the chemical structure of all LATCA compounds used in this study. [file 41588_2022_1052_MOESM5_ESM.zip › 201120_LATCA_Cluster_Structures/2/LAT035E04.png]

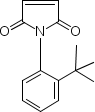

Supplement: Supplementary file 5 — Zip file that contains the chemical structure of all LATCA compounds used in this study. [file 41588_2022_1052_MOESM5_ESM.zip › 201120_LATCA_Cluster_Structures/2/LAT035E11.png]

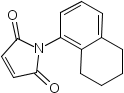

Supplement: Supplementary file 5 — Zip file that contains the chemical structure of all LATCA compounds used in this study. [file 41588_2022_1052_MOESM5_ESM.zip › 201120_LATCA_Cluster_Structures/2/LAT035F03.png]

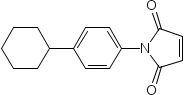

Supplement: Supplementary file 5 — Zip file that contains the chemical structure of all LATCA compounds used in this study. [file 41588_2022_1052_MOESM5_ESM.zip › 201120_LATCA_Cluster_Structures/2/LAT035F04.png]

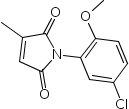

Supplement: Supplementary file 5 — Zip file that contains the chemical structure of all LATCA compounds used in this study. [file 41588_2022_1052_MOESM5_ESM.zip › 201120_LATCA_Cluster_Structures/2/LAT035F08.png]

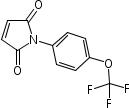

Supplement: Supplementary file 5 — Zip file that contains the chemical structure of all LATCA compounds used in this study. [file 41588_2022_1052_MOESM5_ESM.zip › 201120_LATCA_Cluster_Structures/2/LAT035F09.png]

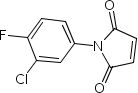

Supplement: Supplementary file 5 — Zip file that contains the chemical structure of all LATCA compounds used in this study. [file 41588_2022_1052_MOESM5_ESM.zip › 201120_LATCA_Cluster_Structures/2/LAT035G07.png]

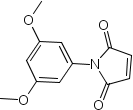

Supplement: Supplementary file 5 — Zip file that contains the chemical structure of all LATCA compounds used in this study. [file 41588_2022_1052_MOESM5_ESM.zip › 201120_LATCA_Cluster_Structures/2/LAT035G08.png]

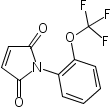

Supplement: Supplementary file 5 — Zip file that contains the chemical structure of all LATCA compounds used in this study. [file 41588_2022_1052_MOESM5_ESM.zip › 201120_LATCA_Cluster_Structures/2/LAT035G09.png]

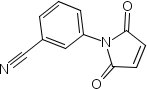

Supplement: Supplementary file 5 — Zip file that contains the chemical structure of all LATCA compounds used in this study. [file 41588_2022_1052_MOESM5_ESM.zip › 201120_LATCA_Cluster_Structures/2/LAT035H04.png]

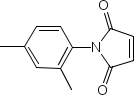

Supplement: Supplementary file 5 — Zip file that contains the chemical structure of all LATCA compounds used in this study. [file 41588_2022_1052_MOESM5_ESM.zip › 201120_LATCA_Cluster_Structures/2/LAT035H07.png]

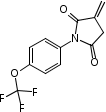

Supplement: Supplementary file 5 — Zip file that contains the chemical structure of all LATCA compounds used in this study. [file 41588_2022_1052_MOESM5_ESM.zip › 201120_LATCA_Cluster_Structures/2/LAT036C02.png]

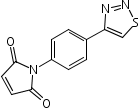

Supplement: Supplementary file 5 — Zip file that contains the chemical structure of all LATCA compounds used in this study. [file 41588_2022_1052_MOESM5_ESM.zip › 201120_LATCA_Cluster_Structures/2/LAT036F03.png]

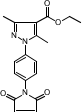

Supplement: Supplementary file 5 — Zip file that contains the chemical structure of all LATCA compounds used in this study. [file 41588_2022_1052_MOESM5_ESM.zip › 201120_LATCA_Cluster_Structures/2/LAT036H07.png]

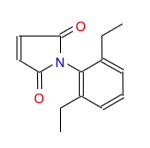

Supplement: Supplementary file 5 — Zip file that contains the chemical structure of all LATCA compounds used in this study. [file 41588_2022_1052_MOESM5_ESM.zip › 201120_LATCA_Cluster_Structures/2/LAT036H11.png]

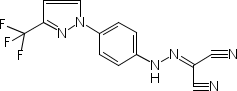

Supplement: Supplementary file 5 — Zip file that contains the chemical structure of all LATCA compounds used in this study. [file 41588_2022_1052_MOESM5_ESM.zip › 201120_LATCA_Cluster_Structures/2/LAT041H04.png]

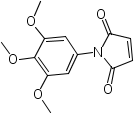

Supplement: Supplementary file 5 — Zip file that contains the chemical structure of all LATCA compounds used in this study. [file 41588_2022_1052_MOESM5_ESM.zip › 201120_LATCA_Cluster_Structures/2/LAT045A04.png]

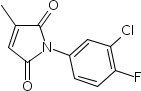

Supplement: Supplementary file 5 — Zip file that contains the chemical structure of all LATCA compounds used in this study. [file 41588_2022_1052_MOESM5_ESM.zip › 201120_LATCA_Cluster_Structures/2/LAT045A06.png]

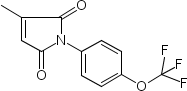

Supplement: Supplementary file 5 — Zip file that contains the chemical structure of all LATCA compounds used in this study. [file 41588_2022_1052_MOESM5_ESM.zip › 201120_LATCA_Cluster_Structures/2/LAT045D07.png]

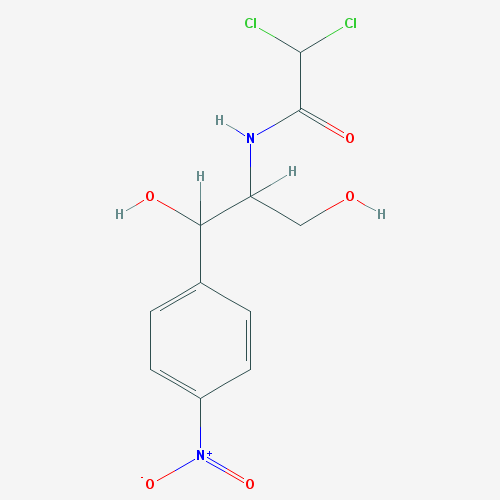

Supplement: Supplementary file 5 — Zip file that contains the chemical structure of all LATCA compounds used in this study. [file 41588_2022_1052_MOESM5_ESM.zip › 201120_LATCA_Cluster_Structures/3/LAT002D07.png]

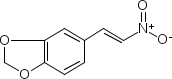

Supplement: Supplementary file 5 — Zip file that contains the chemical structure of all LATCA compounds used in this study. [file 41588_2022_1052_MOESM5_ESM.zip › 201120_LATCA_Cluster_Structures/3/LAT033E09.png]

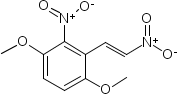

Supplement: Supplementary file 5 — Zip file that contains the chemical structure of all LATCA compounds used in this study. [file 41588_2022_1052_MOESM5_ESM.zip › 201120_LATCA_Cluster_Structures/3/LAT033H07.png]

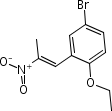

Supplement: Supplementary file 5 — Zip file that contains the chemical structure of all LATCA compounds used in this study. [file 41588_2022_1052_MOESM5_ESM.zip › 201120_LATCA_Cluster_Structures/3/LAT034D11.png]

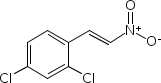

Supplement: Supplementary file 5 — Zip file that contains the chemical structure of all LATCA compounds used in this study. [file 41588_2022_1052_MOESM5_ESM.zip › 201120_LATCA_Cluster_Structures/3/LAT034E07.png]

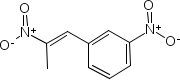

Supplement: Supplementary file 5 — Zip file that contains the chemical structure of all LATCA compounds used in this study. [file 41588_2022_1052_MOESM5_ESM.zip › 201120_LATCA_Cluster_Structures/3/LAT034E11.png]

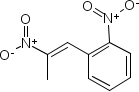

Supplement: Supplementary file 5 — Zip file that contains the chemical structure of all LATCA compounds used in this study. [file 41588_2022_1052_MOESM5_ESM.zip › 201120_LATCA_Cluster_Structures/3/LAT034F11.png]

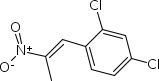

Supplement: Supplementary file 5 — Zip file that contains the chemical structure of all LATCA compounds used in this study. [file 41588_2022_1052_MOESM5_ESM.zip › 201120_LATCA_Cluster_Structures/3/LAT035B07.png]

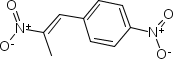

Supplement: Supplementary file 5 — Zip file that contains the chemical structure of all LATCA compounds used in this study. [file 41588_2022_1052_MOESM5_ESM.zip › 201120_LATCA_Cluster_Structures/3/LAT035D07.png]

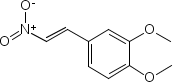

Supplement: Supplementary file 5 — Zip file that contains the chemical structure of all LATCA compounds used in this study. [file 41588_2022_1052_MOESM5_ESM.zip › 201120_LATCA_Cluster_Structures/3/LAT035E03.png]

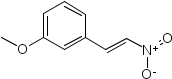

Supplement: Supplementary file 5 — Zip file that contains the chemical structure of all LATCA compounds used in this study. [file 41588_2022_1052_MOESM5_ESM.zip › 201120_LATCA_Cluster_Structures/3/LAT035E07.png]

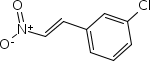

Supplement: Supplementary file 5 — Zip file that contains the chemical structure of all LATCA compounds used in this study. [file 41588_2022_1052_MOESM5_ESM.zip › 201120_LATCA_Cluster_Structures/3/LAT035F02.png]

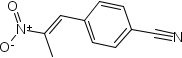

Supplement: Supplementary file 5 — Zip file that contains the chemical structure of all LATCA compounds used in this study. [file 41588_2022_1052_MOESM5_ESM.zip › 201120_LATCA_Cluster_Structures/3/LAT035F05.png]

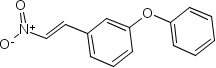

Supplement: Supplementary file 5 — Zip file that contains the chemical structure of all LATCA compounds used in this study. [file 41588_2022_1052_MOESM5_ESM.zip › 201120_LATCA_Cluster_Structures/3/LAT035F10.png]

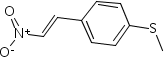

Supplement: Supplementary file 5 — Zip file that contains the chemical structure of all LATCA compounds used in this study. [file 41588_2022_1052_MOESM5_ESM.zip › 201120_LATCA_Cluster_Structures/3/LAT035G05.png]

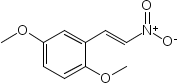

Supplement: Supplementary file 5 — Zip file that contains the chemical structure of all LATCA compounds used in this study. [file 41588_2022_1052_MOESM5_ESM.zip › 201120_LATCA_Cluster_Structures/3/LAT035H06.png]

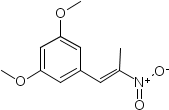

Supplement: Supplementary file 5 — Zip file that contains the chemical structure of all LATCA compounds used in this study. [file 41588_2022_1052_MOESM5_ESM.zip › 201120_LATCA_Cluster_Structures/3/LAT035H08.png]

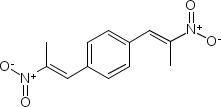

Supplement: Supplementary file 5 — Zip file that contains the chemical structure of all LATCA compounds used in this study. [file 41588_2022_1052_MOESM5_ESM.zip › 201120_LATCA_Cluster_Structures/3/LAT036A03.png]

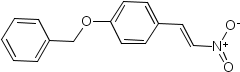

Supplement: Supplementary file 5 — Zip file that contains the chemical structure of all LATCA compounds used in this study. [file 41588_2022_1052_MOESM5_ESM.zip › 201120_LATCA_Cluster_Structures/3/LAT036D02.png]

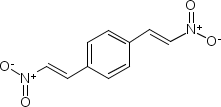

Supplement: Supplementary file 5 — Zip file that contains the chemical structure of all LATCA compounds used in this study. [file 41588_2022_1052_MOESM5_ESM.zip › 201120_LATCA_Cluster_Structures/3/LAT036H02.png]

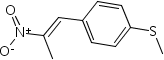

Supplement: Supplementary file 5 — Zip file that contains the chemical structure of all LATCA compounds used in this study. [file 41588_2022_1052_MOESM5_ESM.zip › 201120_LATCA_Cluster_Structures/3/LAT043B09.png]

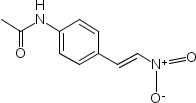

Supplement: Supplementary file 5 — Zip file that contains the chemical structure of all LATCA compounds used in this study. [file 41588_2022_1052_MOESM5_ESM.zip › 201120_LATCA_Cluster_Structures/3/LAT045C08.png]

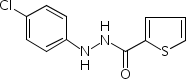

Supplement: Supplementary file 5 — Zip file that contains the chemical structure of all LATCA compounds used in this study. [file 41588_2022_1052_MOESM5_ESM.zip › 201120_LATCA_Cluster_Structures/4/LAT030A09.png]

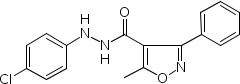

Supplement: Supplementary file 5 — Zip file that contains the chemical structure of all LATCA compounds used in this study. [file 41588_2022_1052_MOESM5_ESM.zip › 201120_LATCA_Cluster_Structures/4/LAT030B09.png]

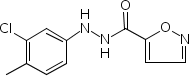

Supplement: Supplementary file 5 — Zip file that contains the chemical structure of all LATCA compounds used in this study. [file 41588_2022_1052_MOESM5_ESM.zip › 201120_LATCA_Cluster_Structures/4/LAT030F07.png]

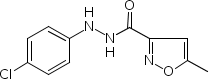

Supplement: Supplementary file 5 — Zip file that contains the chemical structure of all LATCA compounds used in this study. [file 41588_2022_1052_MOESM5_ESM.zip › 201120_LATCA_Cluster_Structures/4/LAT030H08.png]

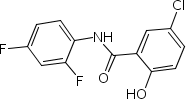

Supplement: Supplementary file 5 — Zip file that contains the chemical structure of all LATCA compounds used in this study. [file 41588_2022_1052_MOESM5_ESM.zip › 201120_LATCA_Cluster_Structures/4/LAT040H08.png]

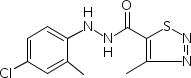

Supplement: Supplementary file 5 — Zip file that contains the chemical structure of all LATCA compounds used in this study. [file 41588_2022_1052_MOESM5_ESM.zip › 201120_LATCA_Cluster_Structures/4/LAT041A08.png]

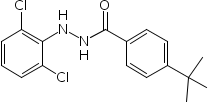

Supplement: Supplementary file 5 — Zip file that contains the chemical structure of all LATCA compounds used in this study. [file 41588_2022_1052_MOESM5_ESM.zip › 201120_LATCA_Cluster_Structures/4/LAT041A11.png]

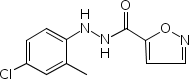

Supplement: Supplementary file 5 — Zip file that contains the chemical structure of all LATCA compounds used in this study. [file 41588_2022_1052_MOESM5_ESM.zip › 201120_LATCA_Cluster_Structures/4/LAT041B08.png]

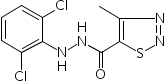

Supplement: Supplementary file 5 — Zip file that contains the chemical structure of all LATCA compounds used in this study. [file 41588_2022_1052_MOESM5_ESM.zip › 201120_LATCA_Cluster_Structures/4/LAT041B11.png]

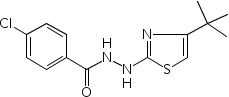

Supplement: Supplementary file 5 — Zip file that contains the chemical structure of all LATCA compounds used in this study. [file 41588_2022_1052_MOESM5_ESM.zip › 201120_LATCA_Cluster_Structures/4/LAT041C06.png]

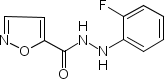

Supplement: Supplementary file 5 — Zip file that contains the chemical structure of all LATCA compounds used in this study. [file 41588_2022_1052_MOESM5_ESM.zip › 201120_LATCA_Cluster_Structures/4/LAT041D09.png]

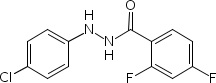

Supplement: Supplementary file 5 — Zip file that contains the chemical structure of all LATCA compounds used in this study. [file 41588_2022_1052_MOESM5_ESM.zip › 201120_LATCA_Cluster_Structures/4/LAT041D10.png]

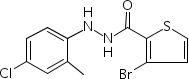

Supplement: Supplementary file 5 — Zip file that contains the chemical structure of all LATCA compounds used in this study. [file 41588_2022_1052_MOESM5_ESM.zip › 201120_LATCA_Cluster_Structures/4/LAT041E08.png]

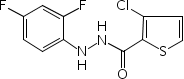

Supplement: Supplementary file 5 — Zip file that contains the chemical structure of all LATCA compounds used in this study. [file 41588_2022_1052_MOESM5_ESM.zip › 201120_LATCA_Cluster_Structures/4/LAT041E10.png]

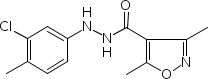

Supplement: Supplementary file 5 — Zip file that contains the chemical structure of all LATCA compounds used in this study. [file 41588_2022_1052_MOESM5_ESM.zip › 201120_LATCA_Cluster_Structures/4/LAT041F07.png]

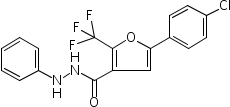

Supplement: Supplementary file 5 — Zip file that contains the chemical structure of all LATCA compounds used in this study. [file 41588_2022_1052_MOESM5_ESM.zip › 201120_LATCA_Cluster_Structures/4/LAT041G06.png]

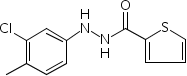

Supplement: Supplementary file 5 — Zip file that contains the chemical structure of all LATCA compounds used in this study. [file 41588_2022_1052_MOESM5_ESM.zip › 201120_LATCA_Cluster_Structures/4/LAT041G07.png]

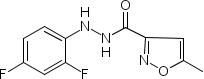

Supplement: Supplementary file 5 — Zip file that contains the chemical structure of all LATCA compounds used in this study. [file 41588_2022_1052_MOESM5_ESM.zip › 201120_LATCA_Cluster_Structures/4/LAT041G10.png]

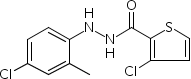

Supplement: Supplementary file 5 — Zip file that contains the chemical structure of all LATCA compounds used in this study. [file 41588_2022_1052_MOESM5_ESM.zip › 201120_LATCA_Cluster_Structures/4/LAT041H07.png]

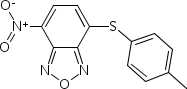

Supplement: Supplementary file 5 — Zip file that contains the chemical structure of all LATCA compounds used in this study. [file 41588_2022_1052_MOESM5_ESM.zip › 201120_LATCA_Cluster_Structures/5/LAT031C11.png]

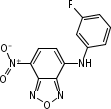

Supplement: Supplementary file 5 — Zip file that contains the chemical structure of all LATCA compounds used in this study. [file 41588_2022_1052_MOESM5_ESM.zip › 201120_LATCA_Cluster_Structures/5/LAT031D11.png]

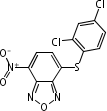

Supplement: Supplementary file 5 — Zip file that contains the chemical structure of all LATCA compounds used in this study. [file 41588_2022_1052_MOESM5_ESM.zip › 201120_LATCA_Cluster_Structures/5/LAT031E11.png]

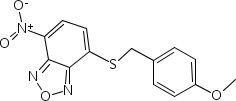

Supplement: Supplementary file 5 — Zip file that contains the chemical structure of all LATCA compounds used in this study. [file 41588_2022_1052_MOESM5_ESM.zip › 201120_LATCA_Cluster_Structures/5/LAT031F11.png]

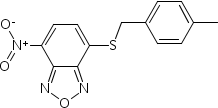

Supplement: Supplementary file 5 — Zip file that contains the chemical structure of all LATCA compounds used in this study. [file 41588_2022_1052_MOESM5_ESM.zip › 201120_LATCA_Cluster_Structures/5/LAT031G11.png]

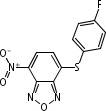

Supplement: Supplementary file 5 — Zip file that contains the chemical structure of all LATCA compounds used in this study. [file 41588_2022_1052_MOESM5_ESM.zip › 201120_LATCA_Cluster_Structures/5/LAT031H11.png]

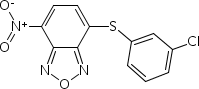

Supplement: Supplementary file 5 — Zip file that contains the chemical structure of all LATCA compounds used in this study. [file 41588_2022_1052_MOESM5_ESM.zip › 201120_LATCA_Cluster_Structures/5/LAT032A02.png]

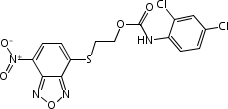

Supplement: Supplementary file 5 — Zip file that contains the chemical structure of all LATCA compounds used in this study. [file 41588_2022_1052_MOESM5_ESM.zip › 201120_LATCA_Cluster_Structures/5/LAT032A03.png]

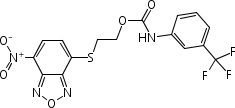

Supplement: Supplementary file 5 — Zip file that contains the chemical structure of all LATCA compounds used in this study. [file 41588_2022_1052_MOESM5_ESM.zip › 201120_LATCA_Cluster_Structures/5/LAT032H02.png]

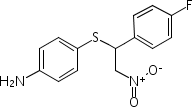

Supplement: Supplementary file 5 — Zip file that contains the chemical structure of all LATCA compounds used in this study. [file 41588_2022_1052_MOESM5_ESM.zip › 201120_LATCA_Cluster_Structures/6/LAT032A11.png]

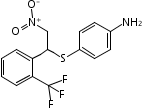

Supplement: Supplementary file 5 — Zip file that contains the chemical structure of all LATCA compounds used in this study. [file 41588_2022_1052_MOESM5_ESM.zip › 201120_LATCA_Cluster_Structures/6/LAT032B11.png]

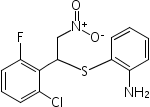

Supplement: Supplementary file 5 — Zip file that contains the chemical structure of all LATCA compounds used in this study. [file 41588_2022_1052_MOESM5_ESM.zip › 201120_LATCA_Cluster_Structures/6/LAT035A06.png]

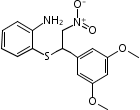

Supplement: Supplementary file 5 — Zip file that contains the chemical structure of all LATCA compounds used in this study. [file 41588_2022_1052_MOESM5_ESM.zip › 201120_LATCA_Cluster_Structures/6/LAT035A09.png]

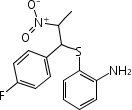

Supplement: Supplementary file 5 — Zip file that contains the chemical structure of all LATCA compounds used in this study. [file 41588_2022_1052_MOESM5_ESM.zip › 201120_LATCA_Cluster_Structures/6/LAT035C07.png]

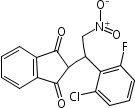

Supplement: Supplementary file 5 — Zip file that contains the chemical structure of all LATCA compounds used in this study. [file 41588_2022_1052_MOESM5_ESM.zip › 201120_LATCA_Cluster_Structures/6/LAT035G06.png]

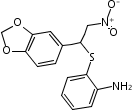

Supplement: Supplementary file 5 — Zip file that contains the chemical structure of all LATCA compounds used in this study. [file 41588_2022_1052_MOESM5_ESM.zip › 201120_LATCA_Cluster_Structures/6/LAT035H05.png]

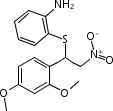

Supplement: Supplementary file 5 — Zip file that contains the chemical structure of all LATCA compounds used in this study. [file 41588_2022_1052_MOESM5_ESM.zip › 201120_LATCA_Cluster_Structures/6/LAT035H09.png]

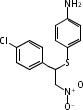

Supplement: Supplementary file 5 — Zip file that contains the chemical structure of all LATCA compounds used in this study. [file 41588_2022_1052_MOESM5_ESM.zip › 201120_LATCA_Cluster_Structures/6/LAT035H10.png]

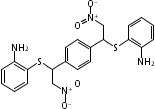

Supplement: Supplementary file 5 — Zip file that contains the chemical structure of all LATCA compounds used in this study. [file 41588_2022_1052_MOESM5_ESM.zip › 201120_LATCA_Cluster_Structures/6/LAT036B03.png]

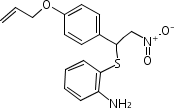

Supplement: Supplementary file 5 — Zip file that contains the chemical structure of all LATCA compounds used in this study. [file 41588_2022_1052_MOESM5_ESM.zip › 201120_LATCA_Cluster_Structures/6/LAT036E02.png]

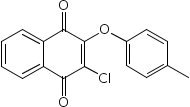

Supplement: Supplementary file 5 — Zip file that contains the chemical structure of all LATCA compounds used in this study. [file 41588_2022_1052_MOESM5_ESM.zip › 201120_LATCA_Cluster_Structures/7/LAT030C11.png]

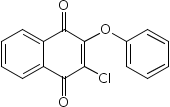

Supplement: Supplementary file 5 — Zip file that contains the chemical structure of all LATCA compounds used in this study. [file 41588_2022_1052_MOESM5_ESM.zip › 201120_LATCA_Cluster_Structures/7/LAT030E11.png]

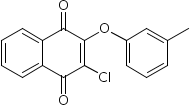

Supplement: Supplementary file 5 — Zip file that contains the chemical structure of all LATCA compounds used in this study. [file 41588_2022_1052_MOESM5_ESM.zip › 201120_LATCA_Cluster_Structures/7/LAT030F11.png]

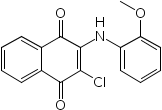

Supplement: Supplementary file 5 — Zip file that contains the chemical structure of all LATCA compounds used in this study. [file 41588_2022_1052_MOESM5_ESM.zip › 201120_LATCA_Cluster_Structures/7/LAT030G11.png]

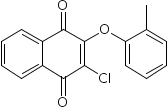

Supplement: Supplementary file 5 — Zip file that contains the chemical structure of all LATCA compounds used in this study. [file 41588_2022_1052_MOESM5_ESM.zip › 201120_LATCA_Cluster_Structures/7/LAT031H02.png]

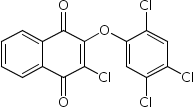

Supplement: Supplementary file 5 — Zip file that contains the chemical structure of all LATCA compounds used in this study. [file 41588_2022_1052_MOESM5_ESM.zip › 201120_LATCA_Cluster_Structures/7/LAT042F03.png]

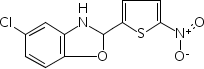

Supplement: Supplementary file 5 — Zip file that contains the chemical structure of all LATCA compounds used in this study. [file 41588_2022_1052_MOESM5_ESM.zip › 201120_LATCA_Cluster_Structures/8/LAT027A05.png]

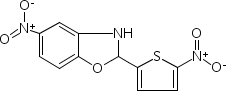

Supplement: Supplementary file 5 — Zip file that contains the chemical structure of all LATCA compounds used in this study. [file 41588_2022_1052_MOESM5_ESM.zip › 201120_LATCA_Cluster_Structures/8/LAT027B05.png]

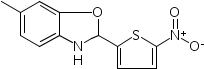

Supplement: Supplementary file 5 — Zip file that contains the chemical structure of all LATCA compounds used in this study. [file 41588_2022_1052_MOESM5_ESM.zip › 201120_LATCA_Cluster_Structures/8/LAT027D05.png]

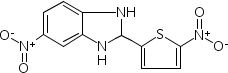

Supplement: Supplementary file 5 — Zip file that contains the chemical structure of all LATCA compounds used in this study. [file 41588_2022_1052_MOESM5_ESM.zip › 201120_LATCA_Cluster_Structures/8/LAT027E05.png]

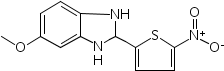

Supplement: Supplementary file 5 — Zip file that contains the chemical structure of all LATCA compounds used in this study. [file 41588_2022_1052_MOESM5_ESM.zip › 201120_LATCA_Cluster_Structures/8/LAT027F05.png]

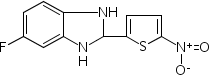

Supplement: Supplementary file 5 — Zip file that contains the chemical structure of all LATCA compounds used in this study. [file 41588_2022_1052_MOESM5_ESM.zip › 201120_LATCA_Cluster_Structures/8/LAT027G05.png]

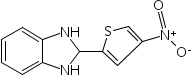

Supplement: Supplementary file 5 — Zip file that contains the chemical structure of all LATCA compounds used in this study. [file 41588_2022_1052_MOESM5_ESM.zip › 201120_LATCA_Cluster_Structures/8/LAT027H04.png]

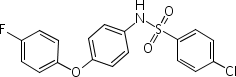

Supplement: Supplementary file 5 — Zip file that contains the chemical structure of all LATCA compounds used in this study. [file 41588_2022_1052_MOESM5_ESM.zip › 201120_LATCA_Cluster_Structures/9/LAT029C11.png]

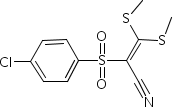

Supplement: Supplementary file 5 — Zip file that contains the chemical structure of all LATCA compounds used in this study. [file 41588_2022_1052_MOESM5_ESM.zip › 201120_LATCA_Cluster_Structures/9/LAT031C06.png]

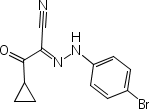

Supplement: Supplementary file 5 — Zip file that contains the chemical structure of all LATCA compounds used in this study. [file 41588_2022_1052_MOESM5_ESM.zip › 201120_LATCA_Cluster_Structures/9/LAT031E05.png]

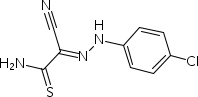

Supplement: Supplementary file 5 — Zip file that contains the chemical structure of all LATCA compounds used in this study. [file 41588_2022_1052_MOESM5_ESM.zip › 201120_LATCA_Cluster_Structures/9/LAT040D07.png]

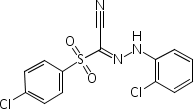

Supplement: Supplementary file 5 — Zip file that contains the chemical structure of all LATCA compounds used in this study. [file 41588_2022_1052_MOESM5_ESM.zip › 201120_LATCA_Cluster_Structures/9/LAT042A08.png]

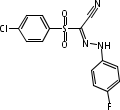

Supplement: Supplementary file 5 — Zip file that contains the chemical structure of all LATCA compounds used in this study. [file 41588_2022_1052_MOESM5_ESM.zip › 201120_LATCA_Cluster_Structures/9/LAT042B08.png]

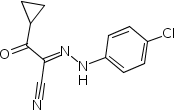

Supplement: Supplementary file 5 — Zip file that contains the chemical structure of all LATCA compounds used in this study. [file 41588_2022_1052_MOESM5_ESM.zip › 201120_LATCA_Cluster_Structures/9/LAT042F07.png]
