# Supplementary figures and images for: Systematic characterization of gene function in the photosynthetic alga Chlamydomonas reinhardtii (part 1 of 2)
Source: Nat Genet. 2022 May 5;54(5):705–14. doi: 10.1038/s41588-022-01052-9 (PMC9110296; doi:10.1038/s41588-022-01052-9)

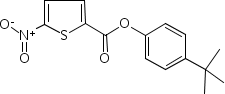

Supplement: Supplementary file 5 — Zip file that contains the chemical structure of all LATCA compounds used in this study. [file 41588_2022_1052_MOESM5_ESM.zip › 201120_LATCA_Cluster_Structures/10/LAT033B06.png]

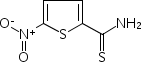

Supplement: Supplementary file 5 — Zip file that contains the chemical structure of all LATCA compounds used in this study. [file 41588_2022_1052_MOESM5_ESM.zip › 201120_LATCA_Cluster_Structures/10/LAT033H05.png]

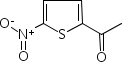

Supplement: Supplementary file 5 — Zip file that contains the chemical structure of all LATCA compounds used in this study. [file 41588_2022_1052_MOESM5_ESM.zip › 201120_LATCA_Cluster_Structures/10/LAT033H10.png]

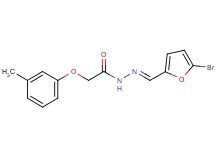

Supplement: Supplementary file 5 — Zip file that contains the chemical structure of all LATCA compounds used in this study. [file 41588_2022_1052_MOESM5_ESM.zip › 201120_LATCA_Cluster_Structures/11/LAT007C10.jpeg]

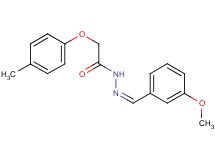

Supplement: Supplementary file 5 — Zip file that contains the chemical structure of all LATCA compounds used in this study. [file 41588_2022_1052_MOESM5_ESM.zip › 201120_LATCA_Cluster_Structures/11/LAT008A05.jpeg]

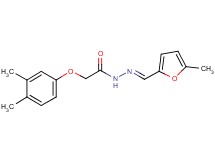

Supplement: Supplementary file 5 — Zip file that contains the chemical structure of all LATCA compounds used in this study. [file 41588_2022_1052_MOESM5_ESM.zip › 201120_LATCA_Cluster_Structures/11/LAT008D07.jpeg]

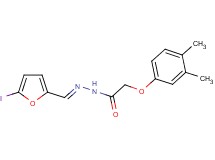

Supplement: Supplementary file 5 — Zip file that contains the chemical structure of all LATCA compounds used in this study. [file 41588_2022_1052_MOESM5_ESM.zip › 201120_LATCA_Cluster_Structures/11/LAT025C04.jpeg]

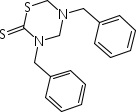

Supplement: Supplementary file 5 — Zip file that contains the chemical structure of all LATCA compounds used in this study. [file 41588_2022_1052_MOESM5_ESM.zip › 201120_LATCA_Cluster_Structures/12/LAT034C08.png]

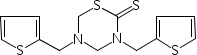

Supplement: Supplementary file 5 — Zip file that contains the chemical structure of all LATCA compounds used in this study. [file 41588_2022_1052_MOESM5_ESM.zip › 201120_LATCA_Cluster_Structures/12/LAT035H03.png]

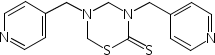

Supplement: Supplementary file 5 — Zip file that contains the chemical structure of all LATCA compounds used in this study. [file 41588_2022_1052_MOESM5_ESM.zip › 201120_LATCA_Cluster_Structures/12/LAT036A02.png]

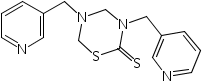

Supplement: Supplementary file 5 — Zip file that contains the chemical structure of all LATCA compounds used in this study. [file 41588_2022_1052_MOESM5_ESM.zip › 201120_LATCA_Cluster_Structures/12/LAT036B02.png]

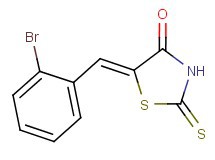

Supplement: Supplementary file 5 — Zip file that contains the chemical structure of all LATCA compounds used in this study. [file 41588_2022_1052_MOESM5_ESM.zip › 201120_LATCA_Cluster_Structures/13/LAT013B05.jpeg]

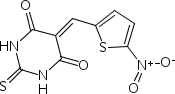

Supplement: Supplementary file 5 — Zip file that contains the chemical structure of all LATCA compounds used in this study. [file 41588_2022_1052_MOESM5_ESM.zip › 201120_LATCA_Cluster_Structures/13/LAT027D04.png]

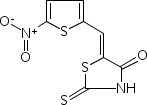

Supplement: Supplementary file 5 — Zip file that contains the chemical structure of all LATCA compounds used in this study. [file 41588_2022_1052_MOESM5_ESM.zip › 201120_LATCA_Cluster_Structures/13/LAT027E04.png]

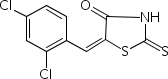

Supplement: Supplementary file 5 — Zip file that contains the chemical structure of all LATCA compounds used in this study. [file 41588_2022_1052_MOESM5_ESM.zip › 201120_LATCA_Cluster_Structures/13/LAT028F03.png]

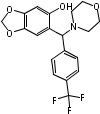

Supplement: Supplementary file 5 — Zip file that contains the chemical structure of all LATCA compounds used in this study. [file 41588_2022_1052_MOESM5_ESM.zip › 201120_LATCA_Cluster_Structures/14/LAT033G04.png]

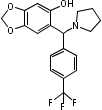

Supplement: Supplementary file 5 — Zip file that contains the chemical structure of all LATCA compounds used in this study. [file 41588_2022_1052_MOESM5_ESM.zip › 201120_LATCA_Cluster_Structures/14/LAT035D10.png]

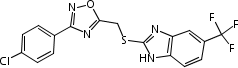

Supplement: Supplementary file 5 — Zip file that contains the chemical structure of all LATCA compounds used in this study. [file 41588_2022_1052_MOESM5_ESM.zip › 201120_LATCA_Cluster_Structures/15/LAT028C11.png]

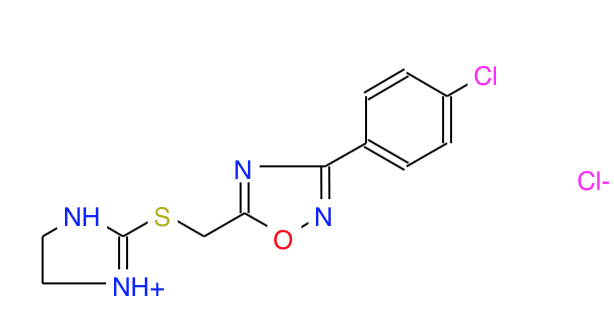

Supplement: Supplementary file 5 — Zip file that contains the chemical structure of all LATCA compounds used in this study. [file 41588_2022_1052_MOESM5_ESM.zip › 201120_LATCA_Cluster_Structures/15/LAT040A06.png]

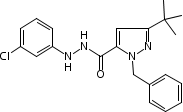

Supplement: Supplementary file 5 — Zip file that contains the chemical structure of all LATCA compounds used in this study. [file 41588_2022_1052_MOESM5_ESM.zip › 201120_LATCA_Cluster_Structures/16/LAT041A10.png]

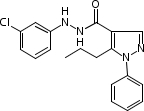

Supplement: Supplementary file 5 — Zip file that contains the chemical structure of all LATCA compounds used in this study. [file 41588_2022_1052_MOESM5_ESM.zip › 201120_LATCA_Cluster_Structures/16/LAT041B10.png]

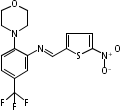

Supplement: Supplementary file 5 — Zip file that contains the chemical structure of all LATCA compounds used in this study. [file 41588_2022_1052_MOESM5_ESM.zip › 201120_LATCA_Cluster_Structures/17/LAT027F04.png]

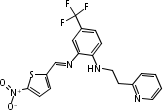

Supplement: Supplementary file 5 — Zip file that contains the chemical structure of all LATCA compounds used in this study. [file 41588_2022_1052_MOESM5_ESM.zip › 201120_LATCA_Cluster_Structures/17/LAT027G04.png]

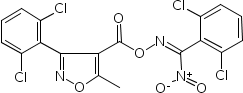

Supplement: Supplementary file 5 — Zip file that contains the chemical structure of all LATCA compounds used in this study. [file 41588_2022_1052_MOESM5_ESM.zip › 201120_LATCA_Cluster_Structures/18/LAT033A06.png]

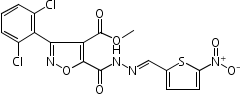

Supplement: Supplementary file 5 — Zip file that contains the chemical structure of all LATCA compounds used in this study. [file 41588_2022_1052_MOESM5_ESM.zip › 201120_LATCA_Cluster_Structures/18/LAT033D06.png]

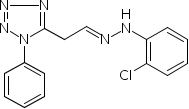

Supplement: Supplementary file 5 — Zip file that contains the chemical structure of all LATCA compounds used in this study. [file 41588_2022_1052_MOESM5_ESM.zip › 201120_LATCA_Cluster_Structures/19/LAT042E05.png]

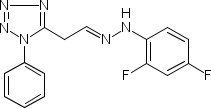

Supplement: Supplementary file 5 — Zip file that contains the chemical structure of all LATCA compounds used in this study. [file 41588_2022_1052_MOESM5_ESM.zip › 201120_LATCA_Cluster_Structures/19/LAT042F05.png]

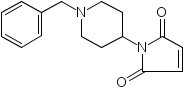

Supplement: Supplementary file 5 — Zip file that contains the chemical structure of all LATCA compounds used in this study. [file 41588_2022_1052_MOESM5_ESM.zip › 201120_LATCA_Cluster_Structures/1/LAT032D10.png]

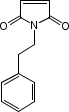

Supplement: Supplementary file 5 — Zip file that contains the chemical structure of all LATCA compounds used in this study. [file 41588_2022_1052_MOESM5_ESM.zip › 201120_LATCA_Cluster_Structures/1/LAT033A02.png]

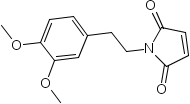

Supplement: Supplementary file 5 — Zip file that contains the chemical structure of all LATCA compounds used in this study. [file 41588_2022_1052_MOESM5_ESM.zip › 201120_LATCA_Cluster_Structures/1/LAT034B07.png]

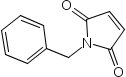

Supplement: Supplementary file 5 — Zip file that contains the chemical structure of all LATCA compounds used in this study. [file 41588_2022_1052_MOESM5_ESM.zip › 201120_LATCA_Cluster_Structures/1/LAT034G07.png]

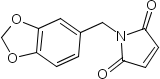

Supplement: Supplementary file 5 — Zip file that contains the chemical structure of all LATCA compounds used in this study. [file 41588_2022_1052_MOESM5_ESM.zip › 201120_LATCA_Cluster_Structures/1/LAT034H10.png]

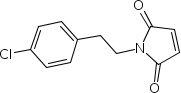

Supplement: Supplementary file 5 — Zip file that contains the chemical structure of all LATCA compounds used in this study. [file 41588_2022_1052_MOESM5_ESM.zip › 201120_LATCA_Cluster_Structures/1/LAT035D05.png]

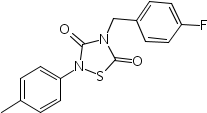

Supplement: Supplementary file 5 — Zip file that contains the chemical structure of all LATCA compounds used in this study. [file 41588_2022_1052_MOESM5_ESM.zip › 201120_LATCA_Cluster_Structures/1/LAT035F11.png]

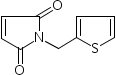

Supplement: Supplementary file 5 — Zip file that contains the chemical structure of all LATCA compounds used in this study. [file 41588_2022_1052_MOESM5_ESM.zip › 201120_LATCA_Cluster_Structures/1/LAT035G03.png]

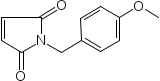

Supplement: Supplementary file 5 — Zip file that contains the chemical structure of all LATCA compounds used in this study. [file 41588_2022_1052_MOESM5_ESM.zip › 201120_LATCA_Cluster_Structures/1/LAT035G11.png]

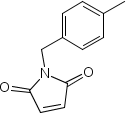

Supplement: Supplementary file 5 — Zip file that contains the chemical structure of all LATCA compounds used in this study. [file 41588_2022_1052_MOESM5_ESM.zip › 201120_LATCA_Cluster_Structures/1/LAT035H11.png]

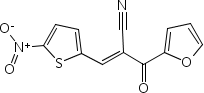

Supplement: Supplementary file 5 — Zip file that contains the chemical structure of all LATCA compounds used in this study. [file 41588_2022_1052_MOESM5_ESM.zip › 201120_LATCA_Cluster_Structures/20/LAT028F11.png]

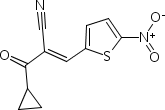

Supplement: Supplementary file 5 — Zip file that contains the chemical structure of all LATCA compounds used in this study. [file 41588_2022_1052_MOESM5_ESM.zip › 201120_LATCA_Cluster_Structures/20/LAT031A06.png]

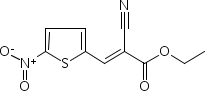

Supplement: Supplementary file 5 — Zip file that contains the chemical structure of all LATCA compounds used in this study. [file 41588_2022_1052_MOESM5_ESM.zip › 201120_LATCA_Cluster_Structures/20/LAT035E02.png]

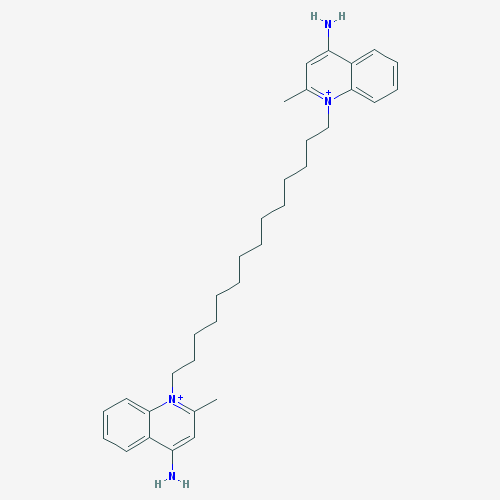

Supplement: Supplementary file 5 — Zip file that contains the chemical structure of all LATCA compounds used in this study. [file 41588_2022_1052_MOESM5_ESM.zip › 201120_LATCA_Cluster_Structures/21/LAT001G11.png]

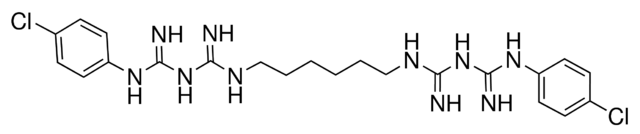

Supplement: Supplementary file 5 — Zip file that contains the chemical structure of all LATCA compounds used in this study. [file 41588_2022_1052_MOESM5_ESM.zip › 201120_LATCA_Cluster_Structures/22/LAT002C07.png]

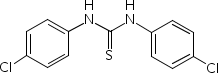

Supplement: Supplementary file 5 — Zip file that contains the chemical structure of all LATCA compounds used in this study. [file 41588_2022_1052_MOESM5_ESM.zip › 201120_LATCA_Cluster_Structures/22/LAT039A06.png]

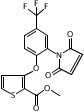

Supplement: Supplementary file 5 — Zip file that contains the chemical structure of all LATCA compounds used in this study. [file 41588_2022_1052_MOESM5_ESM.zip › 201120_LATCA_Cluster_Structures/23/LAT029A11.png]

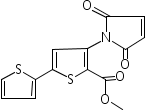

Supplement: Supplementary file 5 — Zip file that contains the chemical structure of all LATCA compounds used in this study. [file 41588_2022_1052_MOESM5_ESM.zip › 201120_LATCA_Cluster_Structures/23/LAT030A05.png]

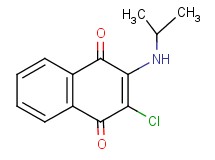

Supplement: Supplementary file 5 — Zip file that contains the chemical structure of all LATCA compounds used in this study. [file 41588_2022_1052_MOESM5_ESM.zip › 201120_LATCA_Cluster_Structures/24/LAT007G11.jpeg]

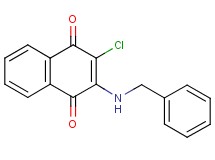

Supplement: Supplementary file 5 — Zip file that contains the chemical structure of all LATCA compounds used in this study. [file 41588_2022_1052_MOESM5_ESM.zip › 201120_LATCA_Cluster_Structures/24/LAT008D04.jpeg]

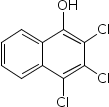

Supplement: Supplementary file 5 — Zip file that contains the chemical structure of all LATCA compounds used in this study. [file 41588_2022_1052_MOESM5_ESM.zip › 201120_LATCA_Cluster_Structures/25/LAT028D05.png]

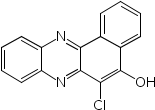

Supplement: Supplementary file 5 — Zip file that contains the chemical structure of all LATCA compounds used in this study. [file 41588_2022_1052_MOESM5_ESM.zip › 201120_LATCA_Cluster_Structures/25/LAT033B03.png]

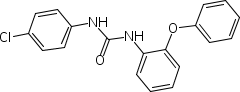

Supplement: Supplementary file 5 — Zip file that contains the chemical structure of all LATCA compounds used in this study. [file 41588_2022_1052_MOESM5_ESM.zip › 201120_LATCA_Cluster_Structures/26/LAT029F03.png]

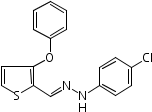

Supplement: Supplementary file 5 — Zip file that contains the chemical structure of all LATCA compounds used in this study. [file 41588_2022_1052_MOESM5_ESM.zip › 201120_LATCA_Cluster_Structures/26/LAT043B04.png]

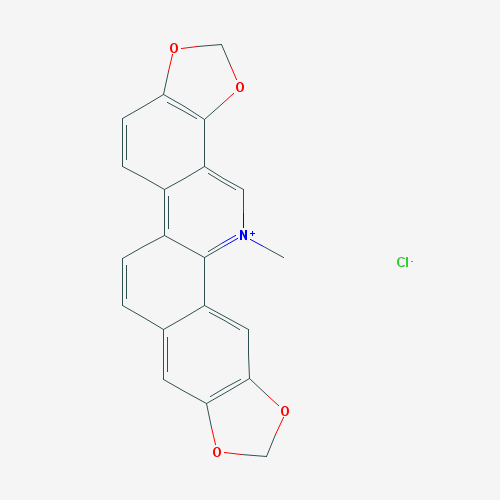

Supplement: Supplementary file 5 — Zip file that contains the chemical structure of all LATCA compounds used in this study. [file 41588_2022_1052_MOESM5_ESM.zip › 201120_LATCA_Cluster_Structures/27/LAT004F06.png]

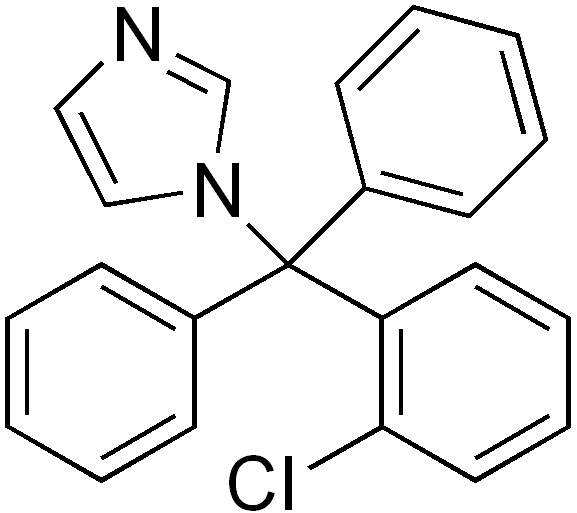

Supplement: Supplementary file 5 — Zip file that contains the chemical structure of all LATCA compounds used in this study. [file 41588_2022_1052_MOESM5_ESM.zip › 201120_LATCA_Cluster_Structures/28/LAT001G07_LAT003D08.png]

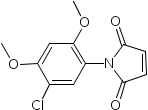

Supplement: Supplementary file 5 — Zip file that contains the chemical structure of all LATCA compounds used in this study. [file 41588_2022_1052_MOESM5_ESM.zip › 201120_LATCA_Cluster_Structures/2/LAT028H08.png]

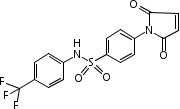

Supplement: Supplementary file 5 — Zip file that contains the chemical structure of all LATCA compounds used in this study. [file 41588_2022_1052_MOESM5_ESM.zip › 201120_LATCA_Cluster_Structures/2/LAT029E10.png]

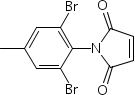

Supplement: Supplementary file 5 — Zip file that contains the chemical structure of all LATCA compounds used in this study. [file 41588_2022_1052_MOESM5_ESM.zip › 201120_LATCA_Cluster_Structures/2/LAT029H10.png]

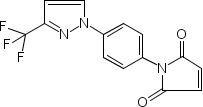

Supplement: Supplementary file 5 — Zip file that contains the chemical structure of all LATCA compounds used in this study. [file 41588_2022_1052_MOESM5_ESM.zip › 201120_LATCA_Cluster_Structures/2/LAT030A04.png]

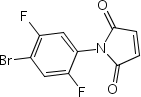

Supplement: Supplementary file 5 — Zip file that contains the chemical structure of all LATCA compounds used in this study. [file 41588_2022_1052_MOESM5_ESM.zip › 201120_LATCA_Cluster_Structures/2/LAT030E03.png]

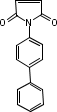

Supplement: Supplementary file 5 — Zip file that contains the chemical structure of all LATCA compounds used in this study. [file 41588_2022_1052_MOESM5_ESM.zip › 201120_LATCA_Cluster_Structures/2/LAT032B10.png]

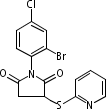

Supplement: Supplementary file 5 — Zip file that contains the chemical structure of all LATCA compounds used in this study. [file 41588_2022_1052_MOESM5_ESM.zip › 201120_LATCA_Cluster_Structures/2/LAT032G11.png]

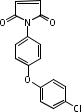

Supplement: Supplementary file 5 — Zip file that contains the chemical structure of all LATCA compounds used in this study. [file 41588_2022_1052_MOESM5_ESM.zip › 201120_LATCA_Cluster_Structures/2/LAT032H11.png]

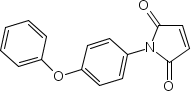

Supplement: Supplementary file 5 — Zip file that contains the chemical structure of all LATCA compounds used in this study. [file 41588_2022_1052_MOESM5_ESM.zip › 201120_LATCA_Cluster_Structures/2/LAT034A08.png]

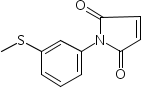

Supplement: Supplementary file 5 — Zip file that contains the chemical structure of all LATCA compounds used in this study. [file 41588_2022_1052_MOESM5_ESM.zip › 201120_LATCA_Cluster_Structures/2/LAT034A09.png]

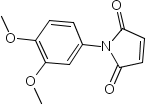

Supplement: Supplementary file 5 — Zip file that contains the chemical structure of all LATCA compounds used in this study. [file 41588_2022_1052_MOESM5_ESM.zip › 201120_LATCA_Cluster_Structures/2/LAT034A11.png]

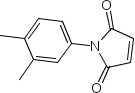

Supplement: Supplementary file 5 — Zip file that contains the chemical structure of all LATCA compounds used in this study. [file 41588_2022_1052_MOESM5_ESM.zip › 201120_LATCA_Cluster_Structures/2/LAT034B08.png]

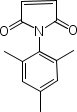

Supplement: Supplementary file 5 — Zip file that contains the chemical structure of all LATCA compounds used in this study. [file 41588_2022_1052_MOESM5_ESM.zip › 201120_LATCA_Cluster_Structures/2/LAT034B09.png]

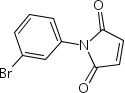

Supplement: Supplementary file 5 — Zip file that contains the chemical structure of all LATCA compounds used in this study. [file 41588_2022_1052_MOESM5_ESM.zip › 201120_LATCA_Cluster_Structures/2/LAT034B11.png]

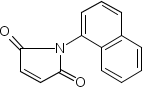

Supplement: Supplementary file 5 — Zip file that contains the chemical structure of all LATCA compounds used in this study. [file 41588_2022_1052_MOESM5_ESM.zip › 201120_LATCA_Cluster_Structures/2/LAT034C07.png]

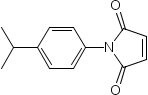

Supplement: Supplementary file 5 — Zip file that contains the chemical structure of all LATCA compounds used in this study. [file 41588_2022_1052_MOESM5_ESM.zip › 201120_LATCA_Cluster_Structures/2/LAT034C09.png]

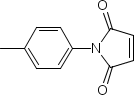

Supplement: Supplementary file 5 — Zip file that contains the chemical structure of all LATCA compounds used in this study. [file 41588_2022_1052_MOESM5_ESM.zip › 201120_LATCA_Cluster_Structures/2/LAT034D06.png]

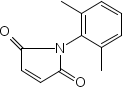

Supplement: Supplementary file 5 — Zip file that contains the chemical structure of all LATCA compounds used in this study. [file 41588_2022_1052_MOESM5_ESM.zip › 201120_LATCA_Cluster_Structures/2/LAT034D07.png]

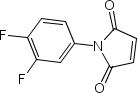

Supplement: Supplementary file 5 — Zip file that contains the chemical structure of all LATCA compounds used in this study. [file 41588_2022_1052_MOESM5_ESM.zip › 201120_LATCA_Cluster_Structures/2/LAT034D10.png]

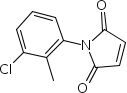

Supplement: Supplementary file 5 — Zip file that contains the chemical structure of all LATCA compounds used in this study. [file 41588_2022_1052_MOESM5_ESM.zip › 201120_LATCA_Cluster_Structures/2/LAT034E06.png]

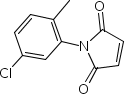

Supplement: Supplementary file 5 — Zip file that contains the chemical structure of all LATCA compounds used in this study. [file 41588_2022_1052_MOESM5_ESM.zip › 201120_LATCA_Cluster_Structures/2/LAT034F06.png]

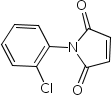

Supplement: Supplementary file 5 — Zip file that contains the chemical structure of all LATCA compounds used in this study. [file 41588_2022_1052_MOESM5_ESM.zip › 201120_LATCA_Cluster_Structures/2/LAT034F07.png]

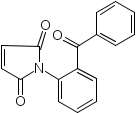

Supplement: Supplementary file 5 — Zip file that contains the chemical structure of all LATCA compounds used in this study. [file 41588_2022_1052_MOESM5_ESM.zip › 201120_LATCA_Cluster_Structures/2/LAT034F10.png]

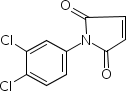

Supplement: Supplementary file 5 — Zip file that contains the chemical structure of all LATCA compounds used in this study. [file 41588_2022_1052_MOESM5_ESM.zip › 201120_LATCA_Cluster_Structures/2/LAT034G06.png]

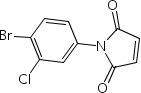

Supplement: Supplementary file 5 — Zip file that contains the chemical structure of all LATCA compounds used in this study. [file 41588_2022_1052_MOESM5_ESM.zip › 201120_LATCA_Cluster_Structures/2/LAT034G08.png]

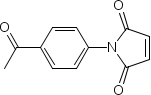

Supplement: Supplementary file 5 — Zip file that contains the chemical structure of all LATCA compounds used in this study. [file 41588_2022_1052_MOESM5_ESM.zip › 201120_LATCA_Cluster_Structures/2/LAT034G09.png]

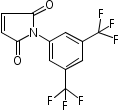

Supplement: Supplementary file 5 — Zip file that contains the chemical structure of all LATCA compounds used in this study. [file 41588_2022_1052_MOESM5_ESM.zip › 201120_LATCA_Cluster_Structures/2/LAT034G11.png]

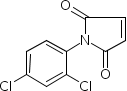

Supplement: Supplementary file 5 — Zip file that contains the chemical structure of all LATCA compounds used in this study. [file 41588_2022_1052_MOESM5_ESM.zip › 201120_LATCA_Cluster_Structures/2/LAT034H06.png]

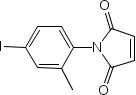

Supplement: Supplementary file 5 — Zip file that contains the chemical structure of all LATCA compounds used in this study. [file 41588_2022_1052_MOESM5_ESM.zip › 201120_LATCA_Cluster_Structures/2/LAT034H07.png]

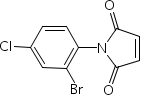

Supplement: Supplementary file 5 — Zip file that contains the chemical structure of all LATCA compounds used in this study. [file 41588_2022_1052_MOESM5_ESM.zip › 201120_LATCA_Cluster_Structures/2/LAT034H08.png]

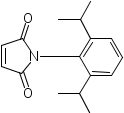

Supplement: Supplementary file 5 — Zip file that contains the chemical structure of all LATCA compounds used in this study. [file 41588_2022_1052_MOESM5_ESM.zip › 201120_LATCA_Cluster_Structures/2/LAT034H11.png]

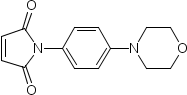

Supplement: Supplementary file 5 — Zip file that contains the chemical structure of all LATCA compounds used in this study. [file 41588_2022_1052_MOESM5_ESM.zip › 201120_LATCA_Cluster_Structures/2/LAT035A02.png]

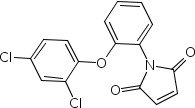

Supplement: Supplementary file 5 — Zip file that contains the chemical structure of all LATCA compounds used in this study. [file 41588_2022_1052_MOESM5_ESM.zip › 201120_LATCA_Cluster_Structures/2/LAT035A05.png]

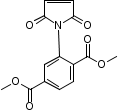

Supplement: Supplementary file 5 — Zip file that contains the chemical structure of all LATCA compounds used in this study. [file 41588_2022_1052_MOESM5_ESM.zip › 201120_LATCA_Cluster_Structures/2/LAT035A10.png]

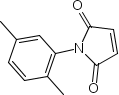

Supplement: Supplementary file 5 — Zip file that contains the chemical structure of all LATCA compounds used in this study. [file 41588_2022_1052_MOESM5_ESM.zip › 201120_LATCA_Cluster_Structures/2/LAT035B02.png]

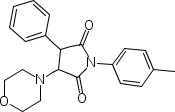

Supplement: Supplementary file 5 — Zip file that contains the chemical structure of all LATCA compounds used in this study. [file 41588_2022_1052_MOESM5_ESM.zip › 201120_LATCA_Cluster_Structures/2/LAT035B08.png]

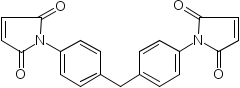

Supplement: Supplementary file 5 — Zip file that contains the chemical structure of all LATCA compounds used in this study. [file 41588_2022_1052_MOESM5_ESM.zip › 201120_LATCA_Cluster_Structures/2/LAT035B09.png]

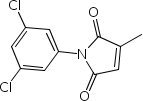

Supplement: Supplementary file 5 — Zip file that contains the chemical structure of all LATCA compounds used in this study. [file 41588_2022_1052_MOESM5_ESM.zip › 201120_LATCA_Cluster_Structures/2/LAT035B10.png]

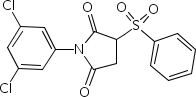

Supplement: Supplementary file 5 — Zip file that contains the chemical structure of all LATCA compounds used in this study. [file 41588_2022_1052_MOESM5_ESM.zip › 201120_LATCA_Cluster_Structures/2/LAT035B11.png]

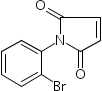

Supplement: Supplementary file 5 — Zip file that contains the chemical structure of all LATCA compounds used in this study. [file 41588_2022_1052_MOESM5_ESM.zip › 201120_LATCA_Cluster_Structures/2/LAT035C02.png]

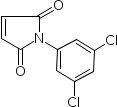

Supplement: Supplementary file 5 — Zip file that contains the chemical structure of all LATCA compounds used in this study. [file 41588_2022_1052_MOESM5_ESM.zip › 201120_LATCA_Cluster_Structures/2/LAT035C03.png]

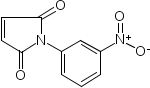

Supplement: Supplementary file 5 — Zip file that contains the chemical structure of all LATCA compounds used in this study. [file 41588_2022_1052_MOESM5_ESM.zip › 201120_LATCA_Cluster_Structures/2/LAT035C09.png]

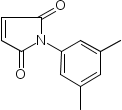

Supplement: Supplementary file 5 — Zip file that contains the chemical structure of all LATCA compounds used in this study. [file 41588_2022_1052_MOESM5_ESM.zip › 201120_LATCA_Cluster_Structures/2/LAT035C11.png]

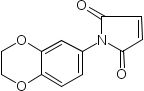

Supplement: Supplementary file 5 — Zip file that contains the chemical structure of all LATCA compounds used in this study. [file 41588_2022_1052_MOESM5_ESM.zip › 201120_LATCA_Cluster_Structures/2/LAT035D02.png]

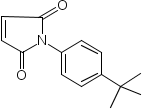

Supplement: Supplementary file 5 — Zip file that contains the chemical structure of all LATCA compounds used in this study. [file 41588_2022_1052_MOESM5_ESM.zip › 201120_LATCA_Cluster_Structures/2/LAT035D03.png]

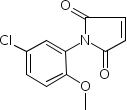

Supplement: Supplementary file 5 — Zip file that contains the chemical structure of all LATCA compounds used in this study. [file 41588_2022_1052_MOESM5_ESM.zip › 201120_LATCA_Cluster_Structures/2/LAT035D08.png]

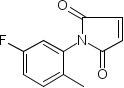

Supplement: Supplementary file 5 — Zip file that contains the chemical structure of all LATCA compounds used in this study. [file 41588_2022_1052_MOESM5_ESM.zip › 201120_LATCA_Cluster_Structures/2/LAT035D09.png]
