# Supplementary material for: Systematic characterization of gene function in the photosynthetic alga Chlamydomonas reinhardtii
Source: Nat Genet. 2022 May 5;54(5):705–14. doi: 10.1038/s41588-022-01052-9 (PMC9110296; doi:10.1038/s41588-022-01052-9)
Supplement: Source Data Fig. 3 — Unprocessed western blots. [file 41588_2022_1052_MOESM7_ESM.pdf]

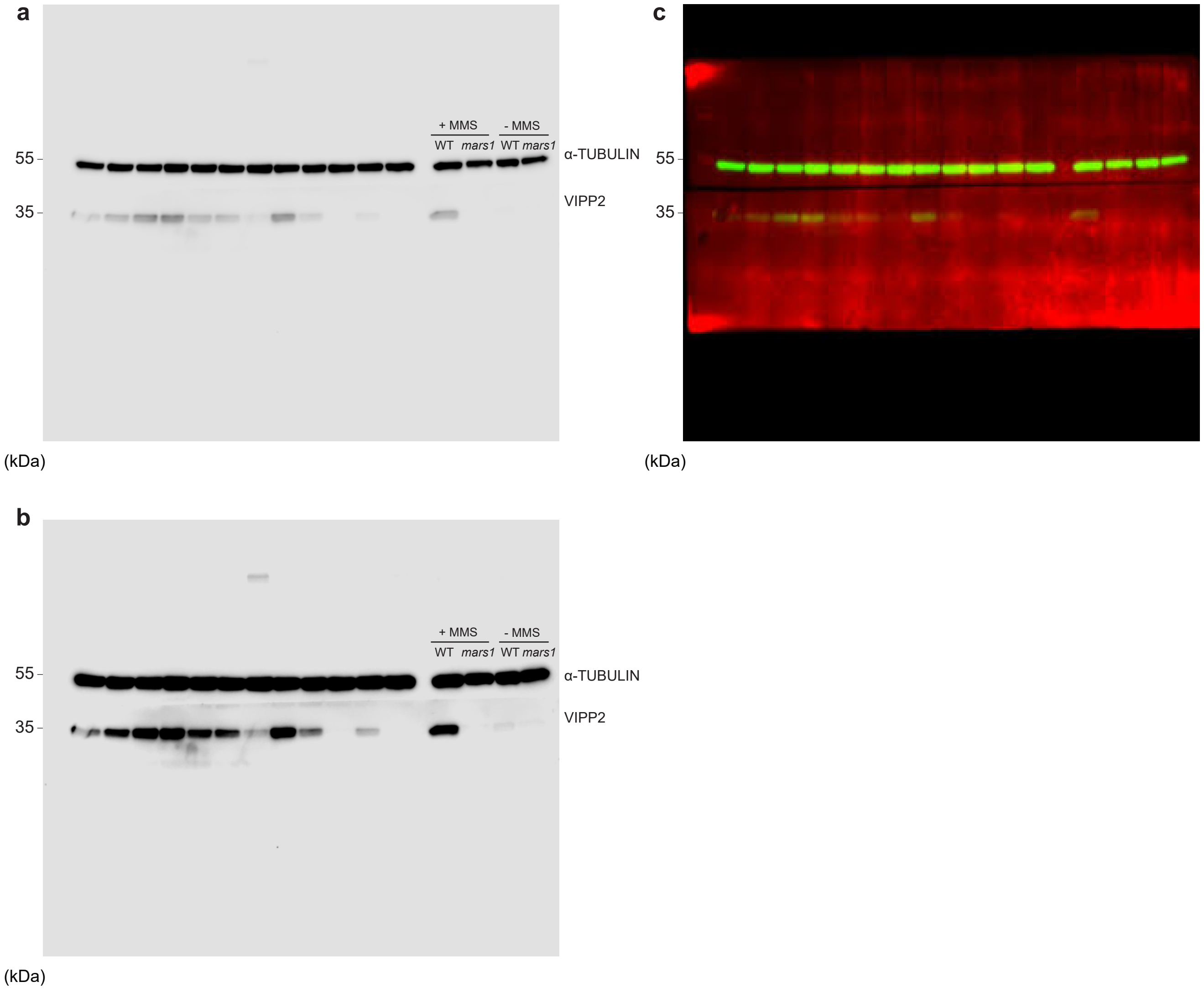

Source Data Figure 3

Immunoblot analysis of VESICLE-INDUCING PROTEIN IN PLASTIDS (VIPP2) found in Figure 3e. The same membrane was cut into two pieces. The upper part was used for the α-TUBULIN blot and the lower part for the VIPP2 blot. A LI-COR imager was used to detect the chemiluminescent signal. ECL channel is displayed. a, light, b, dark. c, Molecular markers (Thermo Scientific PageRuler Plus Prestained Protein Ladder, 26619) were visualized in the fluorescent channels (700 nm) during the detection.
