# Supplementary material for: Systematic characterization of gene function in the photosynthetic alga Chlamydomonas reinhardtii
Source: Nat Genet. 2022 May 5;54(5):705–14. doi: 10.1038/s41588-022-01052-9 (PMC9110296; doi:10.1038/s41588-022-01052-9)
Supplement: Source Data Fig. 5 — Unprocessed western blots. [file 41588_2022_1052_MOESM8_ESM.pdf]

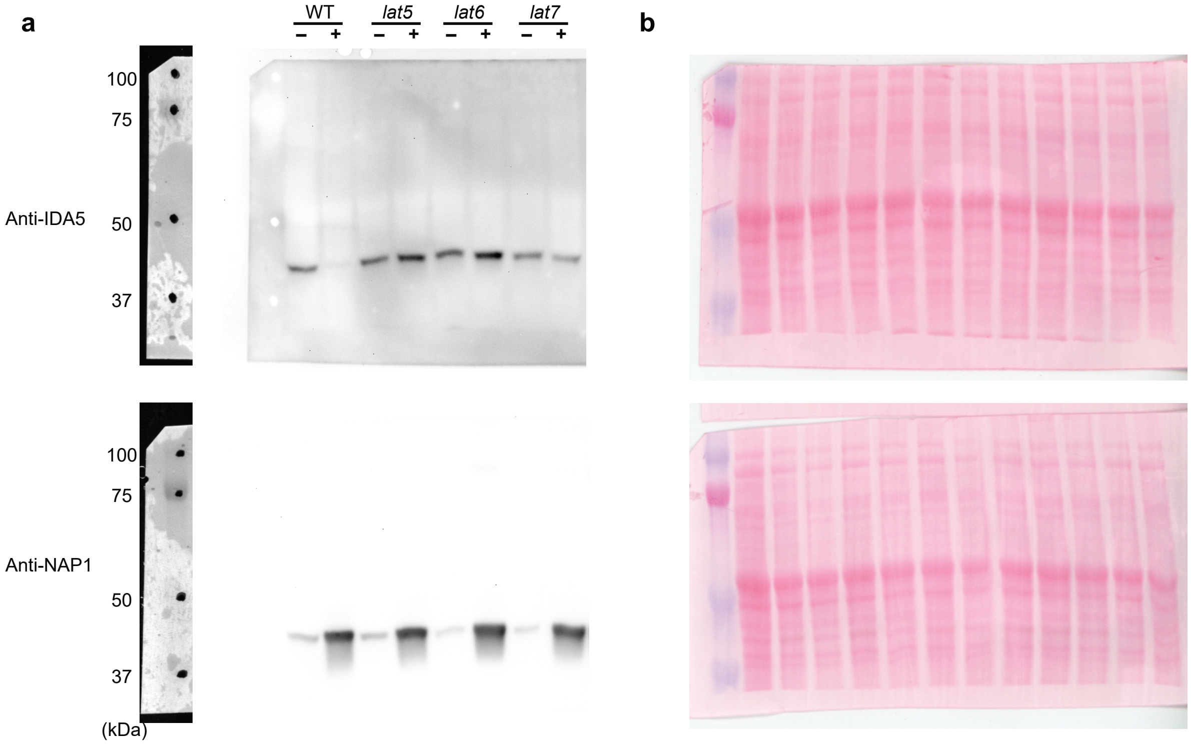


**Source Data Figure 5**

a, Uncropped immunoblot of conventional (IDA5) and alternative (NAP1) actins found in Figure 5e.

b, Uncropped Ponceau stain found in Figure 5e.
